# The inverse palliative care law in advanced lung disease: a mixed-methods systematic review and meta-analysis

**DOI:** 10.1016/j.eclinm.2025.103697

**Published:** 2025-12-17

**Authors:** Donna Wakefield, Tara Dehpour, Clare Bambra, Joanna Davies, Fliss E.M. Murtagh, Jonathan Koffman

**Affiliations:** a Wolfson Palliative Care Research Centre, Hull York Medical School, University of Hull, UK; b Population Health Science Institute, Faculty of Medical Science, Newcastle University, Newcastle-Upon-Tyne, UK; c Cicely Saunders Institute, Department of Palliative Care, Policy and Rehabilitation, Faculty of Nursing, Midwifery and Palliative Care, King's College London, UK

**Keywords:** Inequalities, Lung cancer, Mesothelioma, COPD, ILD, Palliative care

## Abstract

**Background:**

People from socioeconomically deprived backgrounds are at greater risk of developing lung disease and having a higher symptom burden. It remains unclear whether they have equitable access to and experience of palliative care. Therefore, we aimed to synthesise evidence on socioeconomic inequalities in access, receipt of, preference for, and experience of palliative care among people with advanced lung disease.

**Methods:**

Mixed-methods systematic review, searching four databases (MEDLINE, Embase, PsychINFO, CINAHL) from inception to March 28, 2025. We included studies that reported on socioeconomic position and palliative care in advanced lung disease (lung cancer, mesothelioma, chronic obstructive pulmonary disease, interstitial lung disease). Study quality was assessed using the Mixed Methods Appraisal Tool. Both meta-analysis (using a random effects model with I^2^ to assess heterogeneity, sensitivity analysis and GRADE of evidence) and narrative synthesis were performed. PROSPERO CRD42024546502.

**Findings:**

Of 10,572 records, 54 studies met inclusion criteria (4.2 million participants). Meta-analysis of six studies showed people with lung cancer in the lowest SEP group were 18% less likely to receive palliative care than those in the highest (OR 0.82, 95% CI 0.75–0.90, I^2^ = 93.9%). GRADE of evidence was assessed as moderate. Qualitative findings identified financial hardship and insurance barriers limited access to pain relief and oxygen. Few studies considered multiple demographic characteristics, but those that did reported worse access among ethnic minorities and rural populations.

**Interpretation:**

This review provides novel evidence of how the inverse care law operates in advanced lung disease. People from lower socioeconomic groups are significantly less likely to access palliative care, despite greater need. There is an urgent need for equity-focused research and policy interventions, co-produced with underserved communities that account for intersecting social disadvantages.

**Funding:**

10.13039/501100000272National Institute for Health and Care Research.

**Research in context**

**Evidence before this study**

People of lower Socio-Economic Position (SEP) have a higher incidence of malignant and non-malignant lung disease than those of higher socioeconomic position. Previous systematic reviews have identified that despite those of lower SEP having a higher burden of lung disease, they are less likely to receive disease-modifying treatments. For example, those of lower SEP with lung cancer are less likely to receive surgery, chemotherapy or immunotherapy, with a socioeconomic gradient in survival compared to those of higher SEP. These reviews, which are specific to the population with advanced lung disease, focus on inequalities in diagnosis and disease-modifying treatment, with a gap in the evidence on palliative and end-of-life care. Prior systematic reviews have examined the association between socioeconomic position and aspects of end-of-life care (such as hospital use at the end of life and place of death) across the general population. Prior to conducting our systematic review, scoping searches of the literature using MEDLINE from inception until May 2024, identified that no previous reviews had comprehensively synthesised the evidence of socioeconomic inequalities in palliative care, specifically for people with lung disease.

**Added value of this study**

This systematic review is the first to comprehensively synthesise both quantitative and qualitative evidence on the relationship between socioeconomic position and access, receipt and experience of palliative care, specifically for those with advanced lung disease. We provide the first pooled estimates of the association between SEP and receipt of palliative care for those with lung cancer. This meta-analysis identified that people with lung cancer of lower socioeconomic position have reduced odds of receiving palliative care compared to those of higher socioeconomic position, despite a higher level of need, suggesting a palliative care inverse care law operates. Similar findings were seen for those with COPD and ILD, who were also less likely to access palliative care. We also analysed qualitative data, enriching our understanding of the socioeconomic barriers faced and summarised interventions which have been tried to address these barriers. In addition, this is the first review, to our knowledge, to rigorously synthesise how multiple characteristics are recorded within studies, highlighting a critical gap in the current evidence base: as most studies address inequalities related to a single characteristic in isolation without consideration of intersectionality.

**Implications of all the available evidence**

We found consistent evidence that although those of lower socioeconomic position have a higher incidence of advanced lung disease, they are less likely to receive palliative care than those of higher socioeconomic position. We identified significant gaps in the literature, with few studies focused on access to palliative care for those with pleural mesothelioma or interstitial lung disease, a lack of studies on experience of palliative care for those of lower socioeconomic position and lack of consideration of intersectionality. We recommend these areas as a priority for future research.

This work should heighten awareness amongst healthcare professionals, policymakers and the Government that stark inequalities exist for people with lung disease from socioeconomically deprived backgrounds and that equity-focused solutions are needed, tailored to address this. Investment must be made in commissioning services designed to meet the specific needs of patients from low SEP backgrounds and to strive towards delivering equitable palliative care.

## Introduction

People with life-limiting lung disease share a commonality of a progressive illness associated with a high symptom burden.^1^, ^2^, ^3^, ^4^ Non-malignant lung diseases (e.g. chronic obstructive pulmonary disease [COPD], interstitial lung disease [ILD]) are associated with burdensome symptoms and unmet needs similar to those with lung malignancy (lung cancer, pleural mesothelioma), which are challenging to address to improve quality of life.^5^, ^6^, ^7^ Lung cancer is the leading cause of cancer-related death worldwide, accounting for 18% of all cancer deaths globally.^8^ COPD global incidence is estimated to be 480 million cases, projected to increase to 592 million cases by 2050.^9^ Morbidity and mortality from lung disease are not evenly distributed across society. People living in more socioeconomically deprived areas are more likely to experience life-limiting lung disease compared to those in less deprived areas (e.g. 174% increase in the incidence of lung cancer^10^ and double the prevalence of COPD for those living in the most compared to the least deprived areas^11^).

In 1971, Julian Tudor Hart set out The Inverse Care Law: “The availability of good medical care tends to vary inversely with the need for it in the population served. This inverse care law operates more completely where medical care is most exposed to market forces, and less so where such exposure is reduced.”^12^ Evidence suggests that 50 years on, in many healthcare settings, the inverse care law persists.^13^^,^^14^ This is the case for disease-modifying treatment in lung disease. For example, those of lower Socio-Economic Position (SEP) are more likely to develop lung cancer, but they are less likely to receive surgery, chemotherapy or new targeted therapy than those of higher SEP.^15^^,^^16^ Similar inequalities are seen in non-malignant lung disease, for example those with COPD of lower SEP are less likely to access pulmonary rehabilitation.^17^

Misperceptions persist that palliative care is synonymous with end-of-life care and is only available for patients with cancer. Palliative care is an approach that improves quality of life for patients with any life-limiting illness and their families, throughout the disease course.^18^ It is recognised as both a human right^19^ and, in some countries such as the UK, a legal right.^20^ However, evidence suggests that palliative care continues to be underused in COPD and ILD compared to lung cancer.^21^

The majority of palliative care is delivered by non-palliative care specialists, sometimes referred to as “generalist” or “primary” palliative care.^22^ Non-palliative care specialists will be specialists in their own area; who may be based in the community setting (e.g. General Practitioners and community nurses) or hospital-based (e.g. Oncology or Respiratory teams). Non-specialist palliative care providers can deliver the essentials of palliative care such as symptom control, discussing the patient's future wishes and end-of-life care. In more complex situations then support from Specialist Palliative Care teams may be sought, this is a multidisciplinary team who have undertaken further specialist training and are experienced in delivering more complex palliative care. Specialist palliative care can be delivered at home, hospital or hospice. Generally, people would prefer to be cared for at home and avoid hospital admission towards the end of life and so a surrogate marker of high-quality palliative care (non-specialist and specialist) is fewer hospital admissions at the end of life.^23^ People from socioeconomically deprived backgrounds are more likely to be admitted to hospital at the end of life, suggesting inequalities in palliative care.^24^ People from deprived backgrounds are also less likely to receive hospice care.^25^^,^^26^

Lung diseases are more prevalent in deprived communities, carry a heavy symptom burden, and so there are substantial potential benefits from palliative care.^27^, ^28^, ^29^ However, most research to date has focused on inequalities in disease-modifying treatment and survival, rather than palliative care (specialist and non-specialist). Therefore, we aimed to examine whether there is an inverse palliative care law for people with lung disease. Beyond socioeconomic position, other aspects of a person's identity can lead to inequitable access to healthcare, so we aimed to identify how studies report multiple characteristics (e.g. age, gender, ethnicity). We also aimed to summarise studies reporting interventions and evaluated if implementation had the potential to reduce inequalities.

## Methods

### Search strategy and selection criteria

We did a mixed-methods systematic review performed and reported according to the Preferred Reporting Items for Systematic Reviews and Meta-Analysis (PRISMA) criteria. The protocol was registered with PROSPERO (CRD42024546502). The search strategy was developed for MEDLINE (Supplementary Material 1), subsequently adapted for use in other databases. Search filters were identified (e.g. Rietjens^30^ for palliative care terms, Cochrane Register for COPD terms, and Prady^31^ for inequalities terms), plus MeSH and other terms used in previously published reviews. We searched four electronic databases: Ovid MEDLINE, Embase, PsycINFO, CINAHL from inception until the review date (28/03/2025), with no language restrictions.

A mixed-methods synthesis was chosen to combine the generalisability of quantitative data with the contextual depth of qualitative research, providing more comprehensive evidence than either method alone.^32^ Given the review's focus on a broad phenomenon rather than a single outcome, the SPIDER framework^33^ (Sample, Phenomenon of Interest, Design, Evaluation, Research type) was used to guide study selection, as in previous reviews on inequalities.^34^^,^^35^ Our study population (Sample) of interest was adults with advanced lung disease (lung cancer, pleural mesothelioma, Chronic Obstructive Pulmonary Disease, Interstitial Lung Disease), and so we excluded studies that focused on children (<18 years) and/or did not report disaggregated data on advanced lung disease. We included all relevant studies from any geographical region. Our Phenomenon of Interest was socioeconomic position, so we excluded studies which did not include any measure/description of socioeconomic position. We included all study designs, encompassing qualitative, quantitative, and mixed-methods research (e.g. RCTs, cohort studies, focus groups, interviews). We included studies related to access, receipt, preference or experience of any aspect of palliative care (specialist and non-specialist) and excluded studies not related to aspect of palliative care (for example, measures of morbidity/mortality or related to curative treatments only). We excluded studies that did not report primary data (e.g. conference abstracts or protocol only).

### Screening and data extraction

**Fig. 1:**
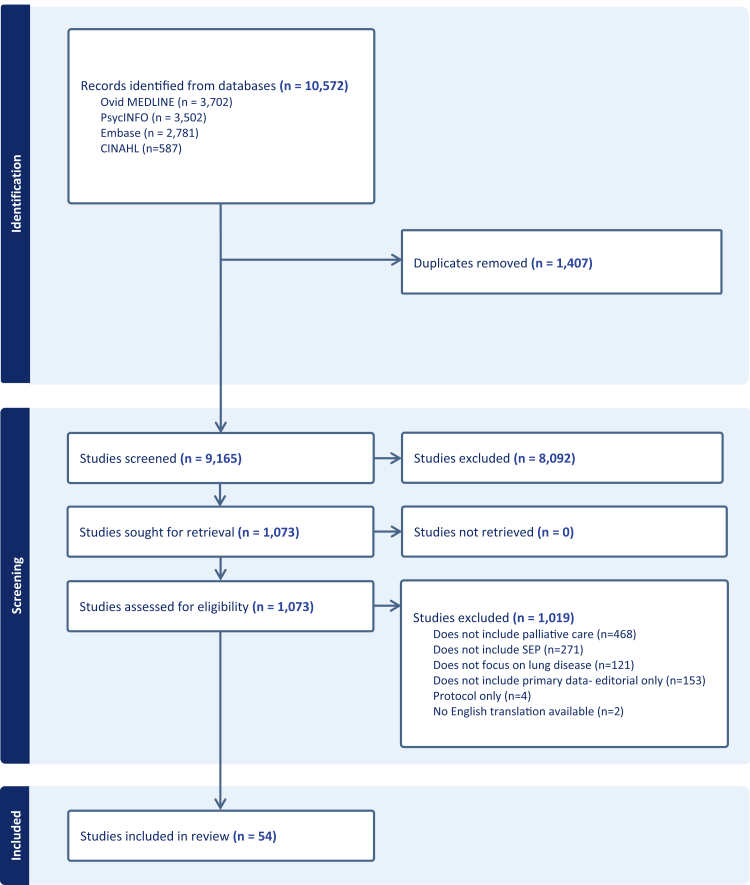
**PRISMA flow chart showing study identification and selection**.

Using EndNote and Covidence, titles and abstracts were screened by one reviewer (DW), 10% (917/9165) independently checked by a second reviewer (TD): inter-rater reliability was found to be high (98%). Where insufficient information was present in the abstract to determine eligibility, full texts were retrieved. Full texts were evaluated by one reviewer (DW), with 10% (108/1073) independently evaluated by TD. Any conflicts were resolved through discussion and adjudication by a third reviewer (JK). This is reported in Fig. 1.

Data extraction included study description (including author, year, setting, title, journal) and data items as per STROBE checklist (STrenghtening the Reporting of Observational studies in Epidemiology) headings (i.e. study design, outcome, exposure variable, methods, analysis, results).^36^ The data extraction was initially piloted for the first five studies and refined as necessary. Data extraction was performed by DW, with 10% (6/54) independently extracted for comparison by TD to ensure validity, with discussion with JK, JD, and FM through the extraction process.

### Data analysis

Study quality was appraised using the Mixed Methods Appraisal Tool (MMAT).^37^ Study quality was assessed based on five questions (Supplementary Material 3) by DW. TD independently reviewed 10% (6/54). Disagreements were resolved through discussion until a consensus was reached. Results from the quality assessment are included in Supplementary Material 2.

**Fig. 2:**
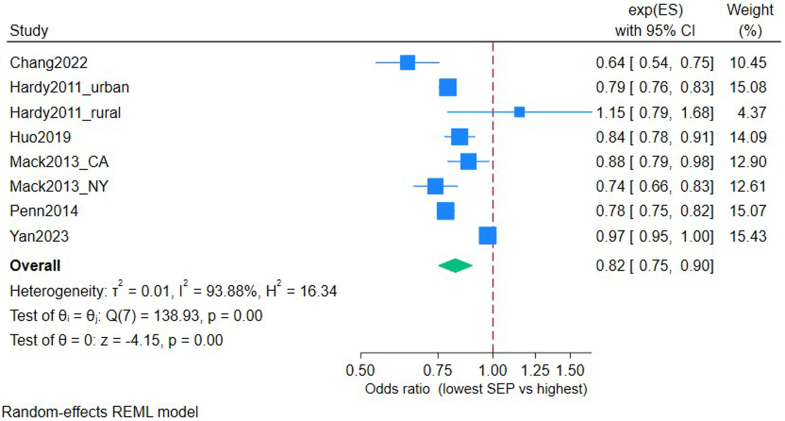
**Forest plot showing the odds of receiving palliative care for people with lung cancer in the lowest area-level Socio-Economic Position (SEP) group compared to those in the highest SEP group.** Multivariable analysis adjusted for: Chang 2022 (age, race, type of insurance, number of comorbidities, illness severity, whether they had surgery, radiotherapy or chemotherapy), Hardy 2011 (age, sex, ethnicity, marital status, tumour stage, comorbidity score, urban/rural), Huo 2019 (age, sex, ethnicity, marital status, rurality, region), Mack 2013 (age at diagnosis, sex, ethnicity, marital status, comorbidities, year of diagnosis, long-term care enrolment), Penn 2014 (age, sex, race, marital status, tumour type), Yan 2023 (age, sex, Elixhauser comorbidity index, rural/urban, diagnosis year).

Data were summarised in a table to enable comparison of included studies describing study type, sample size, exposures and outcomes. For this mixed methods review, we followed the convergent integrated approach recommended by the Joanna Briggs Institute Mixed Methods Group^38^ transforming data into a mutually compatible format to enable synthesis. Data were subsequently pooled within a Microsoft Excel spreadsheet. The pooled data were then categorised into groups based on how they related to the review question and then summarised into a narrative synthesis. In addition, we used Stata v18 to pool odds ratios and 95% confidence intervals across studies reporting comparable outcomes. A priori choice of random-effects model was made with I^2^ to assess heterogeneity (as per Fig. 2). Forest plots were used to visualise between-study variation and overall effects, with a funnel plot to assess the risk of publication bias (Supplementary Material 6), leave-one-out sensitivity analysis and GRADE of evidence were assessed^39^ (Supplementary Material 5).

Three Patient and Public Involvement (PPI) partners with advanced lung disease contributed to shaping the work throughout. They supported the topic, stressing its relevance to inequalities in palliative care access and experiences among those living in deprived areas. While the original proposal focused on lung cancer, PPI partners highlighted that people with non-malignant lung disease often feel “forgotten” and face similar barriers. Based on this feedback, the review's scope was expanded to include both malignant and non-malignant lung diseases.

### Ethics

This is a systematic review of available evidence and is exempt from requiring ethical approval.

### Role of the funding source

The funder of the study had no role in the study design, data collection, data analysis, data interpretation or writing of the report.

## Results

10,572 studies were identified, of which 1407 duplicates were removed. After screening titles and abstracts, 1073 full-text articles were retrieved, of which 54 matched the inclusion criteria (Fig. 1). This included 45 quantitative, five qualitative and four mixed-methods studies. Full description of all included studies was tabulated for transparency^39^, ^40^, ^41^, ^42^, ^43^, ^44^, ^45^, ^46^, ^47^, ^48^, ^49^, ^50^, ^51^, ^52^, ^53^, ^54^, ^55^, ^56^, ^57^, ^58^, ^59^, ^60^, ^61^, ^62^, ^63^, ^64^, ^65^, ^66^, ^67^, ^68^, ^69^, ^70^, ^71^, ^72^, ^73^, ^74^, ^75^, ^76^, ^77^, ^78^, ^79^, ^80^, ^81^, ^82^, ^83^, ^84^, ^85^, ^86^, ^87^, ^88^, ^89^, ^90^, ^91^, ^92^, ^93^ (Supplementary Material 3). The search was not restricted by country, year or language, yet all studies included were published in English between 2003 and 2025 from 11 different countries (USA n = 35, Canada n = 6, UK n = 9, Australia n = 2, Belgium n = 2, France n = 1, Italy n = 1, Sweden n = 1, Taiwan n = 1, Thailand n = 1, New Zealand n = 1).

The review included 4,224,659 people with lung disease, 297 carers and 187 healthcare professionals. Table 1 provides a summary of people with each diagnosis. There was wide variation in how the studies measured socioeconomic position and whether this was at patient level or population level, this is summarised in Table 2.

**Table 1:** Summary of study participants in studies.

Study participants	Number
People with lung cancer	997,057
People with Chronic Obstructive Pulmonary Disease	2,908,258
People with lung cancer AND COPD	24,082
People with Interstitial Lung Disease	295,093
People with Pleural Mesothelioma	169
Carers	297
Healthcare professionals	187
Total	4,225,143

**Table 2:** Summary of how socio-economic position was measured in studies.

	Number of studies
**Individual** level measures n = 20/54 (37%)	
Individual income	4
Individual educational level	7
Individual income AND education	9
**Area-level** measures n = 29/54 (54%)	
Multi-domain area-level deprivation	8
Census (area-level) poverty	5
Area-level income	12
Area-level education	2
Area-level multi-domain deprivation and education	2
**Mix** of individual and area-level measures n = 2/54 (4%)	
Area-level deprivation AND individual-level education	2
Other n = 3/54 (5%)	
Qualitative	3
Total	54

39 studies examined access to or receipt of palliative care. However, only six studies provided comparable or sufficient data to be included in meta-analysis.

A forest plot of six comparable studies is shown in Fig. 2. All included studies used area-level SEP as the exposure and compared receipt of palliative care for people with lung cancer as the outcome, reported as adjusted odds ratios. Two studies^46^^,^^49^ presented two separate estimates based on geography (urban/rural areas and New York/California); these are plotted separately, resulting in 8 estimates overall. The pooled analysis shows the odds of people with lung cancer in the lowest SEP group receiving palliative care to be 0.82 (95% CI 0.75–0.90) compared to the highest SEP group. I^2^ showed high heterogeneity, however, leave-one-out sensitivity analysis (Supplementary Material 5) showed the outcome to be robust with no one paper impacting on effect size. A funnel plot (Supplementary Material 6) was conducted, with a pattern suggesting a small effect size or possible publication bias, although this may be interpreted with caution due to the small number of studies. GRADE^39^ of evidence was applied, defining the certainty of evidence as moderate (Supplementary Material 5). Most remaining studies broadly agreed that low SEP was associated with less receipt of palliative care for those with lung cancer; results are summarised in Table 3.

**Table 3:** Summary of review findings, in relation to objectives.

Domain	Study (author, year)	Lung disease	SEP measure	Key outcome	Direction of inequality	Effect size/insight
Access/Receipt	Mack (2013)^50^	Lung cancer	Area-level income	Hospice receipt	Lower SEP = less access	Lowest SEP = adj OR 0.83; 95% CI 0.77–0.90 (CA), adj OR 0.82; 95% CI 0.75–0.90 (NY)
Access/Receipt	Huo (2019)^53^	Lung cancer	Area-level poverty	SPC receipt	Lower SEP = less SPC	Lowest SEP = adj OR 0.84; 95% CI 0.78–0.91
Access/Receipt	Chang (2022)^56^	Lung cancer	Area-level income	SPC receipt	Lower SEP = less SPC	Lowest SEP = adj OR 0.64; 95% CI 0.54–0.75
Access/Receipt	Ding (2021)^54^	Lung cancer	Area-level deprivation	Inpatient SPC receipt	Lower SEP = less SPC	Lowest SEP = adj OR 0.66; 95% CI 0.55–0.80
Access/Receipt	Penn (2014)^81^	Lung cancer	Education	Hospice access	Lower SEP = less access	Lowest SEP = adj OR 0.78; 95% CI 0.75–0.82
Access/Receipt	Khullar (2022)^57^	Lung cancer	Income & education	SPC receipt	Mixed effect	High income = less access Higher education = more access
Access/Receipt	Simone (2012)^45^	Lung cancer	Education	Pain control	Lower SEP = less access	56% could not afford to take pain medication
Access/Receipt	John (2014)^48^	Lung cancer	Income	Unmet care needs	Lower SEP = more unmet needs	11.4% vs 7.2% (P < 0.001)
Access/Receipt	Saphire (2020)^63^	Lung cancer	Area-level poverty	Pain medication	Lower SEP = less access	Higher SEP = received more meds for pain control
Access/Receipt	Hui (2005)^41^	Lung cancer	Area-level deprivation	SPC receipt	No difference	Binary region comparison, no multivariable analysis
Access/Receipt	Kendzerska (2019)^65^	Lung cancer & COPD	Area-level income	SPC at home	Lower SEP = less SPC	Highest SEP = adj ORs 1.64; 95% CI 1.60–1.68 People with lung cancer are more likely to receive SPC (adj OR 4.22; 95% CI 4.08–4.37) than COPD.
Access/Receipt	Strang (2021)^66^	Lung cancer & COPD	Area-level deprivation	SPC receipt	Lower SEP = less SPC	Highest SEP = adj OR 1.33; 95% CI 1.14–1.56
Access/Receipt	Rush (2017)^68^	COPD	Area-level income	SPC receipt	Lower SEP = less SPC	Highest SEP = adj OR 1.41; 95% CI 1.26–1.58
Access/Receipt	Rush (2018)^77^	ILD	Area-level income	SPC receipt	Lower SEP = less SPC	Highest SEP = adj OR 1.33; 95% CI 0.94–1.
Needs/Preferences	Carlucci (2016)^69^	COPD	Education	EOL treatment preference	Lower SEP = less palliative preference	Lowest SEP = adj OR 15.71 would choose intubation over SPC despite advanced disease
Needs/Preferences	Chou (2017)^72^	COPD	Education	Willingness to accept SPC	Lower SEP = less acceptance	Higher education = higher acceptance of SPC
Needs/Preferences	White (2011)^73^	COPD	Education & area-level deprivation	Hospital readmission preference	Neutral	86/102 patients wanted hospital readmission
Needs/Preferences	Huskamp (2009)^51^	Lung cancer	Income	Discussion of hospice	Lower SEP = less discussion	50% Medicaid vs 69% private insurance
Experience/Outcomes	Cross (2020)^78^	COPD, ILD	Education	Place of death	Lower SEP = more hospital deaths	High SEP = adj OR 1.03; 95% CI 1.01–1.04 (home death) & adj OR 1.19; 95% CI 1.16–1.22 (Hospice death)
Experience/Outcomes	Higginson (2017)^79^	COPD, ILD	Area-level deprivation	Place of death	Lower SEP = more hospital deaths	Dose response: PRs up to 1.55

Abbreviations: SPC, Specialist palliative care; EOL, End-of-life; SEP, Socio-Economic Position; OR, odds ratio; adj, adjusted for covariables; 95% CI, Confidence Interval.

Studies on access to or receipt of palliative care for people with non-malignant lung disease were too heterogeneous for meta-analysis due to variations in methodology—such as qualitative approaches or differing quantitative measures (e.g. place of death, willingness to accept palliative care). Key findings are summarised in Table 4, including evidence that individuals with COPD or ILD from lower socioeconomic groups are less likely to receive palliative care than those from higher SEP groups.

**Table 4:** Studies focussing on potential interventions to improve palliative care in advanced lung disease, which included people of lower Socio-Economic Position (SEP).

Study	Intervention	Study aim	Type	Disease (sample size)	Outcome	Did it reduce inequality?	Impact
Penn^81^ 2014	Ethnic minority focused cancer programme	To identify if patients with lung cancer receiving treatment through a minority-based community oncology programme are more likely to access hospice care.	Outcome-focused evaluation	Lung cancer (76,074)	Black patients receiving treatment at practices enrolled in the minority programme were more likely to receive hospice care. Asian and low-SEP groups still underutilised in hospice.	Partially.	Partial success
Ferrell^85^ 2015	Interdisciplinary palliative care intervention	To test the effectiveness of discussing patients at interdisciplinary care meetings, followed by appropriate referral to supportive care services and four education sessions.	Outcome-focused evaluation	Lung cancer (491)	The intervention improved quality of life and symptom control. Those in the intervention group were more likely to be referred to supportive care. The benefits were greater for those with early-stage disease. People of low SEP were more likely to have late-stage disease at baseline and so potentially less likely to benefit.	Not known. No subgroup analysis between SEP groups.	Success
Nguyen^84^ 2018	Nurse-led community palliative care intervention	To determine the effectiveness of a nurse-led programme (comprehensive patient assessment, interdisciplinary care planning and tailored patient ± caregiver education)	Outcome-focused evaluation	Lung cancer (202)	Most patients enrolled in the study were in the highest income group (>$50,000 USD/annum). The intervention improved patient wellbeing in 3/5 domains (physical, emotional and functional wellbeing). No improvement in distress or reduction in healthcare utilisation.	Not known. No subgroup analysis.	Partial success
Iqbal^86^ 2020	Delphi based referral criteria for outpatient palliative care	Delphi criteria were developed to identify who may benefit from early palliative care referral. This study identified who would be eligible for palliative care based on these criteria and compared this to which patients received palliative care.	Outcome focused evaluation	Lung cancer (38,851)	Those in the lowest income quintile had the highest percentage of patients found to be eligible for SPC using the criteria, i.e. the lower the income, the higher the need for palliative care. Amongst those patients who were eligible for SPC, those in the lowest SEP group had an increased odds of receiving palliative care (lowest SEP group had adj OR 1.13; 95% CI 1.08–1.18 of receiving SPC), compared to the highest SEP group. In contrast to most other studies.	Not the aim of the study	Success
Temel^90^ 2024	Stepped palliative care referral	Early palliative care improves outcomes, but this is unable to be implemented widely due to a lack of workforce. This RCT tested if stepped palliative care (i.e. initial SPC visit, then only subsequent visits if there is a change) is non-inferior	Outcome-focused evaluation	Lung cancer (497) or mesothelioma (10)	Stepped palliative care is non-inferior to automatic early palliative care, which is a more scalable way to deliver palliative care (to overcome workforce limitations).	Not known. No subgroup analysis. Study was inclusive of patients of low SEP.	Success
Horton^83^ 2013	Specialist home palliative care programme- feasibility study	To determine the feasibility of a programme, including education and palliative care follow-up with completion of outcome measures, and it would be feasible to conduct a larger research study to evaluate this.	Feasibility	COPD (30) + caregivers (18)	Challenging to enrol patients within the study period (<60% of target patients enrolled within a year), and completing multiple repeated questionnaires was impractical.	No specific focus on inequalities. The study was inclusive of patients of low SEP.	N/A- Feasibility
Scheerens^74^ 2020	Automatic early integrated palliative home care- feasibility study	To test feasibility, acceptability and preliminary effectiveness of early integrated palliative home care for end-stage COPD	Feasibility	COPD (70)	The early integrated home care was found to be feasible and acceptable to patients. Preliminary results did not find the intervention to be effective for the outcomes (no change in QOL).	No specific focus on inequalities. The study was inclusive of patients of low SEP.	N/A- Feasibility
Reilly^87^ 2023	Online self-guided breathlessness support intervention—feasibility study	To determine whether a randomised controlled trial of “SELF-BREATHE” (a digital support intervention) would be feasible to deliver and acceptable to patients living with chronic breathlessness due to advanced lung disease	Feasibility	COPD (30), ILD (5), Lung cancer (2)	>70% of patients found the intervention and study methods acceptable, supporting moving on to a fully powered RCT to assess efficacy.	No specific focus on inequalities. The study was inclusive of patients of low SEP.	N/A- Feasibility

Qualitative studies contextualise some of the socioeconomic barriers to the receipt of palliative care for patients with COPD. General Practitioners play a vital role in delivering generalist palliative care, yet one (New-Zealand based) study^92^ found that people with a low income were unable to afford the out-of-pocket expenses for a GP appointment, resulting in end-of-life care for COPD being delivered in the hospital emergency department. In COPD, adherence to oxygen improves quality of life and survival; one study^89^ identified that 53% of people had encountered barriers in receiving oxygen due to issues with their insurance. In addition, different types of oxygen delivery exist and people should have optimal devices to meet their needs. People with COPD benefit from maintaining exercise and so oxygen delivery systems, which are smaller, lighter and portable, such as liquid oxygen, are important to enable patients to maintain mobility, improve adherence to oxygen and have a better quality of life.^94^ However, one (USA-based) study highlighted that insurance companies would not pay for liquid oxygen due to higher costs,^89^ “I just list my access to liquid oxygen.....and now I'm on small, compressed gas tanks and it's a different delivery model. So, I'm still adjusting to that, not happily ….but it is what happened to oxygen with the Medicare competitive bid programme”. Patients even struggle to access the inhalers they require due to cost, “I have inhalers. I have the one that's for emergency … And them, my doctor, now gives me samples because they're so expensive.” Healthcare professionals reported that for people with COPD or ILD, palliative care and maintaining quality of life is limited by “what insurance wants to pay and what the patient can ultimately pay too”.^80^

Although lung cancer and COPD are linked to current or past tobacco use, qualitative data revealed that stigma and blame were more commonly directed at people with COPD, which in turn may be a barrier to accessing palliative care.^67^^,^^92^ In most countries, palliative care support is reliant on government and charitable funding, especially for the delivery of hospice care. Participants felt that COPD receives less media attention, partly because it is associated with lower socioeconomic groups, which may in turn negatively affect charitable and government funding for services to deliver more support for patients with COPD.^67^

Four studies^51^^,^^69^^,^^72^^,^^73^ explored preferences of people with COPD in relation to socioeconomic position (SEP). They found that people with advanced COPD and lower educational attainment, a marker of lower SEP, were less likely to accept palliative care and believed it was only available for cancer patients. Although avoiding hospital admission at the end of life is commonly used as a proxy for quality palliative care, structured interviews with 102 people with advanced COPD (all of whom had at least one hospital admission in the past two years) revealed 86 preferred hospital readmissions for future exacerbations, suggesting that avoiding hospitalisation was not their priority.^73^

No studies directly explored variation in quality and patient experience of palliative care related to SEP. Two studies^78^^,^^79^ reported on variation in place of death for people with COPD or ILD. Both showed that those of lower SEP were more likely to die in hospital than those of higher SEP., which will impact on patients’ end-of-life care.

Eight studies examined potential interventions to improve access, receipt or experience of palliative care (including symptom control) for people with lung disease, which included measures of socioeconomic position; these are summarised in Table 4.

**Fig. 3:**
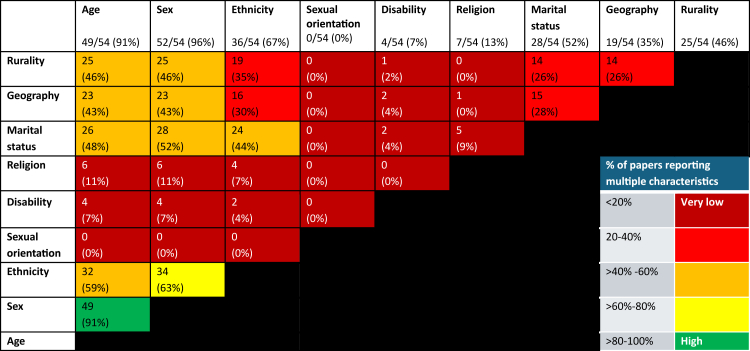
**Summary of studies measuring multiple characteristics**.

Most studies (96%, 52/54) reported participants' sex, typically using a binary classification such as “% male”. None included measures of gender diversity, such as non-binary or transgender identities. Ethnicity was recorded in 36 studies, though reporting varied widely. Six studies reported ethnicity simply as “% White”. An individual's identity is shaped by multiple, overlapping characteristics that collectively influence experiences of disadvantage. The proportion of studies reporting on these multiple characteristics is summarised in Fig. 3.

Only two studies took an intersectional approach to analysis.^46^^,^^54^ In one study, patients were stratified into SEP quartiles and then, within these groups, variation in outcomes was measured according to age, gender, ethnicity, marital status and rurality.^46^ A further study divided patients according to age and gender, then within these groups, variation was examined by SEP, ethnicity and rurality.^54^

## Discussion

We have synthesised specific and novel evidence of how a palliative care ‘inverse care’ law is operating in advanced lung disease. People with lower Socio-Economic Position (SEP) are less likely to receive palliative care, despite having a higher incidence of advanced lung disease and a high symptom burden (Table 3). The strongest evidence of this inverse relationship is for people with lung cancer, with those in the lowest SEP group 18% less likely to receive palliative care than the highest SEP group (Fig. 2). There is also evidence that people with non-malignant lung diseases with lower SEP are similarly disadvantaged; however, far fewer studies have examined inequalities for people with non-malignant lung diseases or mesothelioma, representing a key gap in the literature.

Chronic obstructive pulmonary disease (COPD) and interstitial lung disease (ILD) are life-limiting respiratory illnesses, for which some burdensome symptoms, such as breathlessness, have a higher incidence than lung cancer.^95^ Yet studies identified that those with non-malignant lung disease are less likely to receive palliative care than those with cancer.^21^ Qualitative studies highlighted persistent misperceptions that palliative care is mainly available for people with cancer,^71^ which is just one of the potential barriers to those with non-malignant serious respiratory illness receiving palliative care. While both lung cancer and COPD are strongly linked to current or past smoking, stigma and blame are disproportionately directed at patients with COPD.^67^ COPD is often stereotyped as a “disease of lower socioeconomic class,” and this prejudice and classism may influence not only how welcome those affected feel within healthcare services but also have broader implications for service funding. It is timely that the Lancet commission on palliative care integration in serious respiratory illness has recently been announced, aiming to address this underuse of palliative care in non-malignant lung disease.^96^

Beyond access to palliative care, there is limited evidence on the experience of palliative care received. We found that financial hardship impacts on quality of life and end-of-life care for those with respiratory disease (qualitative data). Most studies were from countries without universal access to healthcare, such as the USA; where the type of health insurance contributed to people having poorer access to hospice care, pain relief, inhalers and oxygen. It is not known how these barriers to palliative care in insurance-based health care systems apply and if they can be generalised to countries with universal access to healthcare. However, recent studies involving people with cancer (not specific to lung disease) have shown that inequalities persist even in universal healthcare systems, with people of lower SEP unable to afford out-of-pocket expenses leading to worse symptom control and poorer quality of life.^97^ Further research is needed to identify how financial hardship affects experience of palliative and end-of-life care for people with all types of respiratory disease in universal healthcare settings and how this can be addressed.

We identified studies showing socioeconomic inequalities in access to palliative care for those with lung disease, as far back as 2003. Two decades later, there is still a lack of rigorous research working with people of lower SEP to identify what services and policies would work for them, to improve their access and experience of palliative care. Eight studies were identified examining interventions to improve access or experience of palliative care for people with lung disease (Table 5), but none of these were specifically focused on tackling socioeconomic inequalities. It is important to recognise that services and policies designed to improve overall access can widen health inequalities further, unless explicitly designed not to do so.^98^ There is a need to adopt proportionate universalism,^99^ with the intensity of research and funding proportionally weighted towards those who are most disadvantaged, to deliver equitable access to high-quality palliative care. The current evidence gap is potentially a result of the perceived methodological barriers when conducting research with people who have both advanced disease and come from lower SEP backgrounds. People of lower SEP are less likely to participate in research. It is vital to be inclusive of these previously underserved groups, with studies tailored to recruit participants from these communities with the highest burden of disease, as failure to do so limits the generalisability of any research findings. A good example of how this can be achieved is in the primary care literature. Similar to the Inverse Care Law (which was initially used to describe poorer access to healthcare in primary care), the term “inverse representation” has been used to describe poorer access to research for those who are disadvantaged. In response to this, general practices at the “Deep End” have led the way in expanding research capacity and capability in areas of high socioeconomic deprivation.^100^ In addition, gatekeeping may occur, where researchers do not offer people with advanced disease the opportunity to take part in research due to concerns that it would be too burdensome. However, studies show that people with advanced disease, regardless of socioeconomic positions, value the opportunity to make their own choice of whether to be involved in research.^101^

All studies included details on socioeconomic position (as part of the search strategy); however, the inclusion of other characteristics within studies varied widely (Fig. 3). Most studies only included this data as covariates, with large variation in covariates used across studies. Some characteristics were reported consistently, such as sex (assigned at birth), while others were not reported at all (such as sexual orientation). Only two studies used multiple characteristics to inform analysis. People face inequalities in access to palliative care related to different aspects of their social identity, for example, those from minority ethnic groups^102^ and LGBTQ+ individuals^103^ are less likely to access palliative care. Intersectionality is the premise that people cannot be reduced to a single characteristic and may experience multiple overlapping layers of disadvantage. Healthcare services are generally designed for those who fit society's norms (white, heterosexual, middle-class, cisgender). It is important to consider intersectionality to gain a more comprehensive understanding of the barriers to palliative care for those with intersecting minoritised identities^104^ and develop a coherent strategy of how to build palliative care services which challenge this. Encouraging researchers to consistently measure multiple characteristics would be the first step in enabling intersectional analyses.

This review has several key strengths. We identified a large number of recent studies without imposing restrictions on country of origin, measures of SEP, or type of palliative care. The inclusion of both quantitative and qualitative evidence enriched the analysis and enhanced the interpretability of findings. A further strength is that to our knowledge, this is the first review to systematically summarise how multiple characteristics are recorded within studies and, crucially, what is missing in studies on socioeconomic inequalities in palliative care. This review highlights a critical gap in the current evidence base: most studies address inequalities related to a single characteristic in isolation. We need more inclusive research designs, with a standardised way of collating inclusive demographic data, to capture complex identities and disadvantage. Only by doing so can we develop a deeper, more accurate understanding of inequalities and ultimately improve palliative care for underserved populations.

This review has several limitations. A study methodology limitation was the use of single screening and quality assessment, with only a proportion independently checked by a second reviewer. Using a complete dual review process has the potential to increase the number of relevant studies identified, but this is resource-intensive and was not feasible with the size of this review. When agreement between reviewers is strong, then this method provides similar results and is a pragmatic alternative, used widely in the literature.^105^^,^^106^ A further limitation was the small number of comparable studies for meta-analysis, and high heterogeneity. In addition, there was a lack of in-depth qualitative data, with data on ILD and mesothelioma particularly scarce. Although the review was not restricted to high-income countries, all included studies originated from such settings. This absence of data from low- and middle-income countries represents a significant gap in the evidence base. Future research should therefore prioritise the inclusion of low- and middle-income countries to help identify context-specific challenges and to support the global effort to reduce inequities in palliative care access and quality.

This review highlights a stark and persistent inequity: those most in need of palliative care for advanced lung disease, especially people from socioeconomically disadvantaged backgrounds, are consistently least likely to receive it. Despite two decades of evidence, progress remains limited, with few interventions designed explicitly to reduce these inequalities. Future research must move beyond describing disparities and instead focus on co-producing, implementing, and evaluating equity-focused solutions that are acceptable, scalable, and tailored to communities with the highest unmet needs. This includes designing interventions that consider people's complex, intersecting identities, recognising that socioeconomic status alone does not explain exclusion. To address the inverse palliative care law, the Government, funders, researchers, clinicians and policymakers must invest in inclusive research, systematically capture multiple demographic characteristics, and embed intersectionality within both research and practice. Only then can we design and deliver palliative care services that are truly equitable and responsive to the needs of those currently most underserved.

## Contributors

Conceptualisation DW, JK, FM, JD.

2nd Reviewer TD.

Data curation DW.

Formal analysis DW, JD.

Project administration DW.

Supervision, validation JK, FM, JD, CB.

Writing—original draft DW.

Review & editing- All.

DW and JD accessed and verified the study data.

All authors have read and approved the final version of the manuscript.

## Data sharing statement

This mixed-methods systematic review and meta-analysis is based on published, peer-reviewed manuscripts.

## Declaration of interests

We declare no competing interests.

## Appendix A Supplementary data

MEDLINE Search Terms

Quality Assessment

Copy of MMAT questions

Details of all included studies

Sensitivity analysis and GRADE

Funnel plot
