# Supplementary material for: The inverse palliative care law in advanced lung disease: a mixed-methods systematic review and meta-analysis
Source: eClinicalMedicine. 2025 Dec 17;91:103697. doi: 10.1016/j.eclinm.2025.103697 (PMC12770954; doi:10.1016/j.eclinm.2025.103697)
Supplement: MEDLINE Search Terms [file mmc1.docx]

**Supplementary material 1**

**Ovid MEDLINE Search terms**

1. Palliative Care search terms

| **Query** |  |
| --- | --- |
| 1 | exp advance care planning/ |
| 2 | exp attitude to death/ |
| 3 | exp bereavement/ |
| 4 | death/ |
| 5 | hospices/ or "Hospice and Palliative Care Nursing"/ |
| 6 | life support care/ |
| 7 | palliative care/ or Palliative Medicine/ |
| 8 | exp terminal care/ or respite care/ |
| 9 | terminally ill/ |
| 10 | palliat$.af. |
| 11 | hospice$.af. |
| 12 | (terminal care or respite care).af. |
| 13 | or/1-12 |
| 14 | journal of palliative care.jn. |
| 15 | journal of palliative medicine.jn. |
| 16 | hospice journal physical psychosocial & pastoral care of the dying.jn. |
| 17 | supportive care in cancer.jn. |
| 18 | palliative medicine.jn. |
| 19 | palliative & supportive care.jn. |
| 20 | journal of supportive oncology.jn. |
| 21 | journal of social work in end of life & palliative care.jn. |
| 22 | journal of pain & symptom management.jn. |
| 23 | journal of pain & palliative care pharmacotherapy.jn. |
| 24 | international journal of palliative nursing.jn. |
| 25 | death studies.jn. |
| 26 | death education.jn. |
| 27 | american journal of hospice care.jn. |
| 28 | american journal of hospice & palliative medicine.jn. |
| 29 | omega journal of death & dying.jn. |
| 30 | or/14-29 |
| 31 | 13 or 30 |
| 32 | bereave*.mp. |
| 33 | attitude to death.mp. |
| 34 | end of life.af. |
| 35 | Advance* Care.af. |
| 36 | ((advanced or terminal*) adj (ill* or disease)).ti,ab,kw. |
| 37 | supportive care.ti,ab,kw. |
| 38 | dying.ti,ab,kw. |
| 39 | "last year of life".ti,ab,kw. |
| 40 | (limited life adj (expectanc* or span*)).ti,ab,kw. or life-limiting.mp. |
| 41 | or/32-40 |
| 42 | 31 or 41 [palliative care concept] |

1. **Chronic Obstructive Disease** (COPD) search terms

| 43 | exp "Lung Diseases, Obstructive"/ |
| --- | --- |
| 44 | emphysema$.mp. |
| 45 | COPD.mp. |
| 46 | AECB.mp. |
| 47 | COBD.mp. |
| 48 | COAD.mp. |
| 49 | (obstruct$ adj3 (pulmonary or lungs$ or airway$ or airflow$ or bronch$ or respirat$)).mp. |
| 50 | (chronic$ adj3 bronchiti$).mp. |
| 51 | 43 or 44 or 45 or 46 or 47 or 48 or 49 or 50 [ based on COPD Cochrane register search strategy] |

1. **Interstitial Lung Disease** (ILD) search terms

| 52 | exp "Lung Diseases, Interstitial"/ |
| --- | --- |
| 53 | exp pulmonary fibrosis/ |
| 54 | (interstitial$ adj3 (lung$ adj3 disease$)).tw. |
| 55 | (interstitial$ adj3 (fibros$ or pneumonitis or pneumonia or pneumopathy)).tw. |
| 56 | (diffuse* adj3 parenchymal*).tw. |
| 57 | alveolitis.mp. |
| 58 | exp Bronchiolitis Obliterans/ or (bronchiolitis adj obliterans).mp. |
| 59 | (goodpasture$ adj syndrome$).mp. |
| 60 | granulomatosis.mp. |
| 61 | exp Histiocytosis/ or histiocytosis$.mp. |
| 62 | exp Pneumoconiosis/ or pneumoconiosis.mp. or pneumokoniosis.mp. or pneumonoconiosis.mp. |
| 63 | bagassosis.mp. |
| 64 | (pulmonary$ adj sarcoid$).mp. |
| 65 | (pulmonary$ adj fibros$).mp. |
| 66 | (wegener$ adj granuloma$).mp. |
| 67 | (lung$ adj purpura).mp. |
| 68 | ((bird$ or farmer$ or pigeon$ or avian$ or budgerigar$) adj (lung$ or disease$)).mp. |
| 69 | (asbestosis or byssinosis or siderosis or silicosis or berylliosis or anthracosilicosis or silicotuberculosis).mp. |
| 70 | 52 or 53 or 54 or 55 or 56 or 57 or 58 or 59 or 60 or 61 or 62 or 63 or 64 or 65 or 66 or 67 or 68 or 69 [ILD search terms- based on ESR review] |

1. **Lung cancer** search terms

| 71 | exp "Lung Neoplasm"/ |
| --- | --- |
| 72 | exp "Pleural Neoplasms"/ |
| 73 | exp bronchial neoplasms/ |
| 74 | pancoast syndrome.mp. [mp=title, book title, abstract, original title, name of substance word, subject heading word, floating sub-heading word, keyword heading word, organism supplementary concept word, protocol supplementary concept word, rare disease supplementary concept word, unique identifier, synonyms, population supplementary concept word, anatomy supplementary concept word] |
| 75 | lung cancer*.mp. [mp=title, book title, abstract, original title, name of substance word, subject heading word, floating sub-heading word, keyword heading word, organism supplementary concept word, protocol supplementary concept word, rare disease supplementary concept word, unique identifier, synonyms, population supplementary concept word, anatomy supplementary concept word] |
| 76 | Bronchogenic carcinoma*.mp. |
| 77 | Non-Small-Cell Lung ca$.mp. [mp=title, book title, abstract, original title, name of substance word, subject heading word, floating sub-heading word, keyword heading word, organism supplementary concept word, protocol supplementary concept word, rare disease supplementary concept word, unique identifier, synonyms, population supplementary concept word, anatomy supplementary concept word] |
| 78 | NSCLC.mp. [mp=title, book title, abstract, original title, name of substance word, subject heading word, floating sub-heading word, keyword heading word, organism supplementary concept word, protocol supplementary concept word, rare disease supplementary concept word, unique identifier, synonyms, population supplementary concept word, anatomy supplementary concept word] |
| 79 | SCLC.mp. |
| 80 | lung adenocarcinoma*.mp. [mp=title, book title, abstract, original title, name of substance word, subject heading word, floating sub-heading word, keyword heading word, organism supplementary concept word, protocol supplementary concept word, rare disease supplementary concept word, unique identifier, synonyms, population supplementary concept word, anatomy supplementary concept word] |
| 81 | Large cell carcinoma*.mp. [mp=title, book title, abstract, original title, name of substance word, subject heading word, floating sub-heading word, keyword heading word, organism supplementary concept word, protocol supplementary concept word, rare disease supplementary concept word, unique identifier, synonyms, population supplementary concept word, anatomy supplementary concept word] |
| 82 | Small cell carcinoma*.mp. [mp=title, book title, abstract, original title, name of substance word, subject heading word, floating sub-heading word, keyword heading word, organism supplementary concept word, protocol supplementary concept word, rare disease supplementary concept word, unique identifier, synonyms, population supplementary concept word, anatomy supplementary concept word] |
| 83 | Bronchiolo-Alveolar.mp. [mp=title, book title, abstract, original title, name of substance word, subject heading word, floating sub-heading word, keyword heading word, organism supplementary concept word, protocol supplementary concept word, rare disease supplementary concept word, unique identifier, synonyms, population supplementary concept word, anatomy supplementary concept word] |
| 84 | Bronchioloalveolar.mp. [mp=title, book title, abstract, original title, name of substance word, subject heading word, floating sub-heading word, keyword heading word, organism supplementary concept word, protocol supplementary concept word, rare disease supplementary concept word, unique identifier, synonyms, population supplementary concept word, anatomy supplementary concept word] |
| 85 | mesothelioma.mp. [mp=title, book title, abstract, original title, name of substance word, subject heading word, floating sub-heading word, keyword heading word, organism supplementary concept word, protocol supplementary concept word, rare disease supplementary concept word, unique identifier, synonyms, population supplementary concept word, anatomy supplementary concept word] |
| 86 | 71 or 72 or 73 or 74 or 75 or 76 or 77 or 78 or 79 or 80 or 81 or 82 or 83 or 84 or 85 [lung cancers] |

Combined searches

| 87 | 51 or 70 or 86 ALL lung disease terms (COPD or ILD or lung cancer) |
| --- | --- |
| 88 | 42 and 87 All lung disease AND palliative care terms |

1. Inequalities/inequities/lack of access general search terms

| 89 | Health Status Disparities/ |
| --- | --- |
| 90 | Health Services Access$.mp. [mp=title, book title, abstract, original title, name of substance word, subject heading word, floating sub-heading word, keyword heading word, organism supplementary concept word, protocol supplementary concept word, rare disease supplementary concept word, unique identifier, synonyms, population supplementary concept word, anatomy supplementary concept word] |
| 91 | Health Equity/ |
| 92 | health*care disparit*.mp. |
| 93 | health care disparit*.mp. |
| 94 | health status disparit*.mp. |
| 95 | health disparit*.mp. |
| 96 | health inequalit*.mp. |
| 97 | health inequit*.mp. |
| 98 | Medically Underserved Area/ or underserved.mp. |
| 99 | (access adj5 (fair or unfair or fairness or unfairness)).tw. |
| 100 | (barrier$ adj5 (treatment$ or care)).tw. |
| 101 | 89 or 90 or 91 or 92 or 93 or 94 or 95 or 96 or 97 or 98 or 99 or 100 |
|  | 88 and 101 (lung disease + palliative care + general inequalities/access) =240 |

1. Socio-economic status and poverty related inequalities/inequities

| 102 | Social determinants of Health/ |
| --- | --- |
| 103 | Psychosocial Deprivation/ |
| 104 | Sociological Factors/ |
| 105 | Working Poor/ |
| 106 | Hierarchy, Social/ |
| 107 | disparit*.mp. |
| 108 | inequalit*.mp. |
| 109 | inequit*.mp. |
| 110 | equity.mp. |
| 111 | deprivation |
| 112 | gini.mp. |
| 113 | concentration index.mp. |
| 114 | Socioeconomic Factors/ |
| 115 | Social Welfare/ |
| 116 | exp Social Class/ |
| 117 | exp Poverty/ |
| 118 | Income/ |
| 119 | Social class*.mp. |
| 120 | social determinants.mp. |
| 121 | social status.mp. |
| 122 | social position.mp. |
| 123 | social background.mp. |
| 124 | social circumstance*.mp. |
| 125 | socio-economic.mp. |
| 126 | socioeconomic.mp. |
| 127 | sociodemographic.mp. |
| 128 | socio-demographic.mp. |
| 129 | SES.mp. |
| 130 | disadvantaged.mp. |
| 131 | impoverished.mp. |
| 132 | poverty.mp. |
| 133 | economic level.mp. |
| 134 | assets index.mp. |
| 135 | occupation.mp. |
| 136 | uneducated.mp. |
| 137 | low education.mp. |
| 138 | social* disadvantage*.mp. |
| 139 | employment*.tw. |
| 140 | (level adj2 education*).tw. |
| 141 | literate*.tw. |
| 142 | literac*.tw. |
| 143 | welfare*.tw. |
| 144 | post?code.tw. |
| 145 | post code.tw. |
| 146 | neighbourhood*.tw. |
| 147 | exp Public Assistance/ |
| 148 | 102 or 103 or 104 or 105 or 106 or 107 or 108 or 109 or 110 or 111 or 112 or 113 or 114 or 115 or 116 or 117 or 118 or 119 or 120 or 121 or 122 or 123 or 124 or 125 or 126 or 127 or 128 or 129 or 130 or 131 or 132 or 133 or 134 or 135 or 136 or 137 or 138 or 139 or 140 or 141 or 142 or 143 or 144 or 145 or 146 or 147 |

Combined searches

| 149 | 101 or 148 (general inequalities/lack of access AND SES related inequalities combined) |
| --- | --- |
| 150 | 88 and 149 (lung disease and palliative care and all inequalities terms) |

Removed terms:

income*.mp.= 206983 results and often irrelevant as mentioned high/middle/low income setting

benefit*.tw.= 917631 too many results related to other things like “benefit of treatment_
