# Supplementary material for: The inverse palliative care law in advanced lung disease: a mixed-methods systematic review and meta-analysis
Source: eClinicalMedicine. 2025 Dec 17;91:103697. doi: 10.1016/j.eclinm.2025.103697 (PMC12770954; doi:10.1016/j.eclinm.2025.103697)
Supplement: Quality Assessment [file mmc2.docx]

**Supplementary material 2:**

**Quality assessment using Mixed Methods Appraisal Tool**

| **Studies** |  | | **Criteria from the Mixed Methods Appraisal Tool** | | | | | | | | | | | | | | | | | | | | | | | | | | |
| --- | --- | --- | --- | --- | --- | --- | --- | --- | --- | --- | --- | --- | --- | --- | --- | --- | --- | --- | --- | --- | --- | --- | --- | --- | --- | --- | --- | --- | --- |
|  | S1 | S2 | | 1.1 | 1.2 | 1.3 | 1.4 | 1.5 | 2.1 | 2.2 | 2.3 | 2.4 | 2.5 | 3.1 | 3.2 | 3.3 | 3.4 | 3.5 | 4.1 | 4.2 | 4.3 | 4.4 | 4.5 | 5.1 | 5.2 | 5.3 | 5.4 | 5.5 | Overall (% of quality criteria met) |
| Vranas (2020) | Y | Y | |  |  |  |  |  |  |  |  |  |  | Y | Y | Y | Y | Y |  |  |  |  |  |  |  |  |  |  | 100% |
| Hui  (2005) | Y | Y | |  |  |  |  |  |  |  |  |  |  | Y | N | Y | N | Y |  |  |  |  |  |  |  |  |  |  | 60% |
| Davidoff  (2021) | Y | Y | |  |  |  |  |  |  |  |  |  |  | Y | Y | Y | Y | Y |  |  |  |  |  |  |  |  |  |  | 100% |
| Sullivan  (2017) | Y | Y | |  |  |  |  |  |  |  |  |  |  | Y | Y | Y | Y | Y |  |  |  |  |  |  |  |  |  |  | 100% |
| McCarthy  (2003) | Y | Y | |  |  |  |  |  |  |  |  |  |  | Y | Y | Y | Y | Y |  |  |  |  |  |  |  |  |  |  | 100% |
| Simone  (2011) | Y | Y | |  |  |  |  |  |  |  |  |  |  |  |  |  |  |  | Y | N | Y | N | Y |  |  |  |  |  | 60% |
| Yan  (2023) | Y | Y | |  |  |  |  |  |  |  |  |  |  | Y | Y | Y | Y | Y |  |  |  |  |  |  |  |  |  |  | 100% |
| Hardy  (2011) | Y | Y | |  |  |  |  |  |  |  |  |  |  | Y | Y | Y | Y | Y |  |  |  |  |  |  |  |  |  |  | 100% |
| John  (2014) | Y | Y | |  |  |  |  |  |  |  |  |  |  |  |  |  |  |  | Y | Y | Y | Y | Y |  |  |  |  |  | 100% |
| Bylicki  (2021) | Y | Y | |  |  |  |  |  |  |  |  |  |  | Y | Y | N | Y | Y |  |  |  |  |  |  |  |  |  |  | 80% |
| Mack  (2013) | Y | Y | |  |  |  |  |  |  |  |  |  |  | Y | Y | Y | Y | Y |  |  |  |  |  |  |  |  |  |  | 100% |
| Huskamp  (2009) | Y | Y | |  |  |  |  |  |  |  |  |  |  | Y | Y | Y | Y | Y |  |  |  |  |  |  |  |  |  |  | 100% |
| Keating  (2006) | Y | Y | |  |  |  |  |  |  |  |  |  |  | Y | Y | Y | N | Y |  |  |  |  |  |  |  |  |  |  | 80% |
| Huo  (2019) | Y | Y | |  |  |  |  |  |  |  |  |  |  | Y | Y | Y | Y | Y |  |  |  |  |  |  |  |  |  |  | 100% |
| Ding  (2021) | Y | Y | |  |  |  |  |  |  |  |  |  |  | Y | Y | Y | Y | Y |  |  |  |  |  |  |  |  |  |  | 100% |
| Shugarnan  (2007) | Y | Y | |  |  |  |  |  |  |  |  |  |  | Y | Y | Y | Y | Y |  |  |  |  |  |  |  |  |  |  | 100% |
| Chang  (2022) | Y | Y | |  |  |  |  |  |  |  |  |  |  | Y | Y | Y | Y | Y |  |  |  |  |  |  |  |  |  |  | 100% |
| Khullar  (2022) | Y | Y | |  |  |  |  |  |  |  |  |  |  | Y | Y | Y | Y | Y |  |  |  |  |  |  |  |  |  |  | 100% |
| Nayar  (2014) | Y | Y | |  |  |  |  |  |  |  |  |  |  | Y | Y | Y | Y | Y |  |  |  |  |  |  |  |  |  |  | 100% |
| McLouth  (2023) | Y | Y | |  |  |  |  |  |  |  |  |  |  |  |  |  |  |  | Y | Y | N | Y | N |  |  |  |  |  | 60% |
| Goldie  (2021) | Y | Y | |  |  |  |  |  |  |  |  |  |  | Y | Y | Y | Y | Y |  |  |  |  |  |  |  |  |  |  | 100% |
| Schweiger  (2023) | Y | Y | |  |  |  |  |  |  |  |  |  |  | Y | Y | Y | Y | Y |  |  |  |  |  |  |  |  |  |  | 100% |
| Huo  (2021) | Y | Y | |  |  |  |  |  |  |  |  |  |  | Y | Y | Y | Y | Y |  |  |  |  |  |  |  |  |  |  | 100% |
| Saphire  (2020) | Y | Y | |  |  |  |  |  |  |  |  |  |  | Y | Y | Y | Y | Y |  |  |  |  |  |  |  |  |  |  | 100% |
| Burt  (2010) | Y | Y | |  |  |  |  |  |  |  |  |  |  |  |  |  |  |  | Y | Y | Y | N | Y |  |  |  |  |  | 80% |
| Kendzerska  (2019) | Y | Y | |  |  |  |  |  |  |  |  |  |  | Y | Y | Y | Y | Y |  |  |  |  |  |  |  |  |  |  | 100% |
| Strang  (2021) | Y | Y | |  |  |  |  |  |  |  |  |  |  | Y | Y | Y | Y | Y |  |  |  |  |  |  |  |  |  |  | 100% |
| Fairlamb  (2021) | Y | Y | | Y | Y | Y | Y | Y |  |  |  |  |  |  |  |  |  |  |  |  |  |  |  |  |  |  |  |  | 100% |
| Rush  (2017) | Y | Y | |  |  |  |  |  |  |  |  |  |  | Y | Y | Y | Y | Y |  |  |  |  |  |  |  |  |  |  | 100% |
| Carlucci  (2016) | Y | Y | |  |  |  |  |  |  |  |  |  |  | Y | Y | Y | Y | Y |  |  |  |  |  |  |  |  |  |  | 100% |
| Knauft  (2005) | Y | Y | |  |  |  |  |  |  |  |  |  |  |  |  |  |  |  |  |  |  |  |  | N | Y | Y | Y | N | 60% |
| Fu  (2021) | Y | Y | | Y | Y | Y | Y | Y |  |  |  |  |  |  |  |  |  |  |  |  |  |  |  |  |  |  |  |  | 100% |
| Chia-Wen  (2017) | Y | Y | |  |  |  |  |  |  |  |  |  |  | N | Y | Y | Y | Y |  |  |  |  |  |  |  |  |  |  | 80% |
| White  (2011) | Y | Y | | Y | Y | Y | Y | Y |  |  |  |  |  |  |  |  |  |  |  |  |  |  |  |  |  |  |  |  | 100% |
| Scheerens  (2020) | Y | Y | |  |  |  |  |  |  |  |  |  |  | Y | Y | Y | Y | Y |  |  |  |  |  |  |  |  |  |  | 100% |
| Gershon  (2018) | Y | Y | |  |  |  |  |  |  |  |  |  |  | Y | Y | N | N | Y |  |  |  |  |  |  |  |  |  |  | 60% |
| Sono  (2023) | Y | Y | |  |  |  |  |  |  |  |  |  |  | N | N | Y | N | Y |  |  |  |  |  |  |  |  |  |  | 40% |
| Rush  (2018) | Y | Y | |  |  |  |  |  |  |  |  |  |  | Y | Y | Y | Y | Y |  |  |  |  |  |  |  |  |  |  | 100% |
| Cross  (2020) | Y | Y | |  |  |  |  |  |  |  |  |  |  | Y | Y | Y | Y | Y |  |  |  |  |  |  |  |  |  |  | 100% |
| Higginson  (2017) | Y | Y | |  |  |  |  |  |  |  |  |  |  | Y | Y | Y | Y | Y |  |  |  |  |  |  |  |  |  |  | 100% |
| McVeigh  (2019) | Y | Y | | Y | Y | Y | Y | Y |  |  |  |  |  |  |  |  |  |  |  |  |  |  |  |  |  |  |  |  | 100% |
| Penn  (2014) | Y | Y | |  |  |  |  |  |  |  |  |  |  | Y | Y | Y | Y | Y |  |  |  |  |  |  |  |  |  |  | 100% |
| Scheerens  (2020) | Y | Y | |  |  |  |  |  | Y | N | Y | N | N |  |  |  |  |  |  |  |  |  |  |  |  |  |  |  | 40% |
| Horton  (2013) | Y | Y | |  |  |  |  |  |  |  |  |  |  | Y | Y | N | N | Y |  |  |  |  |  |  |  |  |  |  | 60% |
| Nguyen  (2018) | Y | Y | |  |  |  |  |  |  |  |  |  |  | N | Y | N | Y | Y |  |  |  |  |  |  |  |  |  |  | 60% |
| Ferrell  (2015) | Y | Y | |  |  |  |  |  |  |  |  |  |  | N | Y | Y | Y | Y |  |  |  |  |  |  |  |  |  |  | 80% |
| Iqbal  (2020) | Y | Y | |  |  |  |  |  |  |  |  |  |  | Y | Y | Y | Y | Y |  |  |  |  |  |  |  |  |  |  | 100% |
| Reilly  (2023) | Y | Y | |  |  |  |  |  | Y | Y | N | Y | Y |  |  |  |  |  |  |  |  |  |  |  |  |  |  |  | 80% |
| Armstrong  (2025) | Y | Y | |  |  |  |  |  |  |  |  |  |  | Y | Y | Y | N | Y |  |  |  |  |  |  |  |  |  |  | 80% |
| Madiraca  (2024) | Y | Y | |  |  |  |  |  |  |  |  |  |  |  |  |  |  |  |  |  |  |  |  | N | N | N | Y | Y | 40% |
| Temel  (2024) | Y | Y | |  |  |  |  |  | Y | Y | Y | N | Y |  |  |  |  |  |  |  |  |  |  |  |  |  |  |  | 80% |
| Edmonds  (2025) | Y | Y | |  |  |  |  |  |  |  |  |  |  | N | Y | Y | Y | Y |  |  |  |  |  |  |  |  |  |  | 80% |
| Landers  (2024) | Y | Y | | Y | Y | Y | Y | Y |  |  |  |  |  |  |  |  |  |  |  |  |  |  |  |  |  |  |  |  | 100% |
| Wakefield  (2025) | Y | Y | |  |  |  |  |  |  |  |  |  |  | Y | Y | Y | N | Y |  |  |  |  |  |  |  |  |  |  | 80% |
