# Supplementary figures and images for: The inverse palliative care law in advanced lung disease: a mixed-methods systematic review and meta-analysis
Source: eClinicalMedicine. 2025 Dec 17;91:103697. doi: 10.1016/j.eclinm.2025.103697 (PMC12770954; doi:10.1016/j.eclinm.2025.103697)

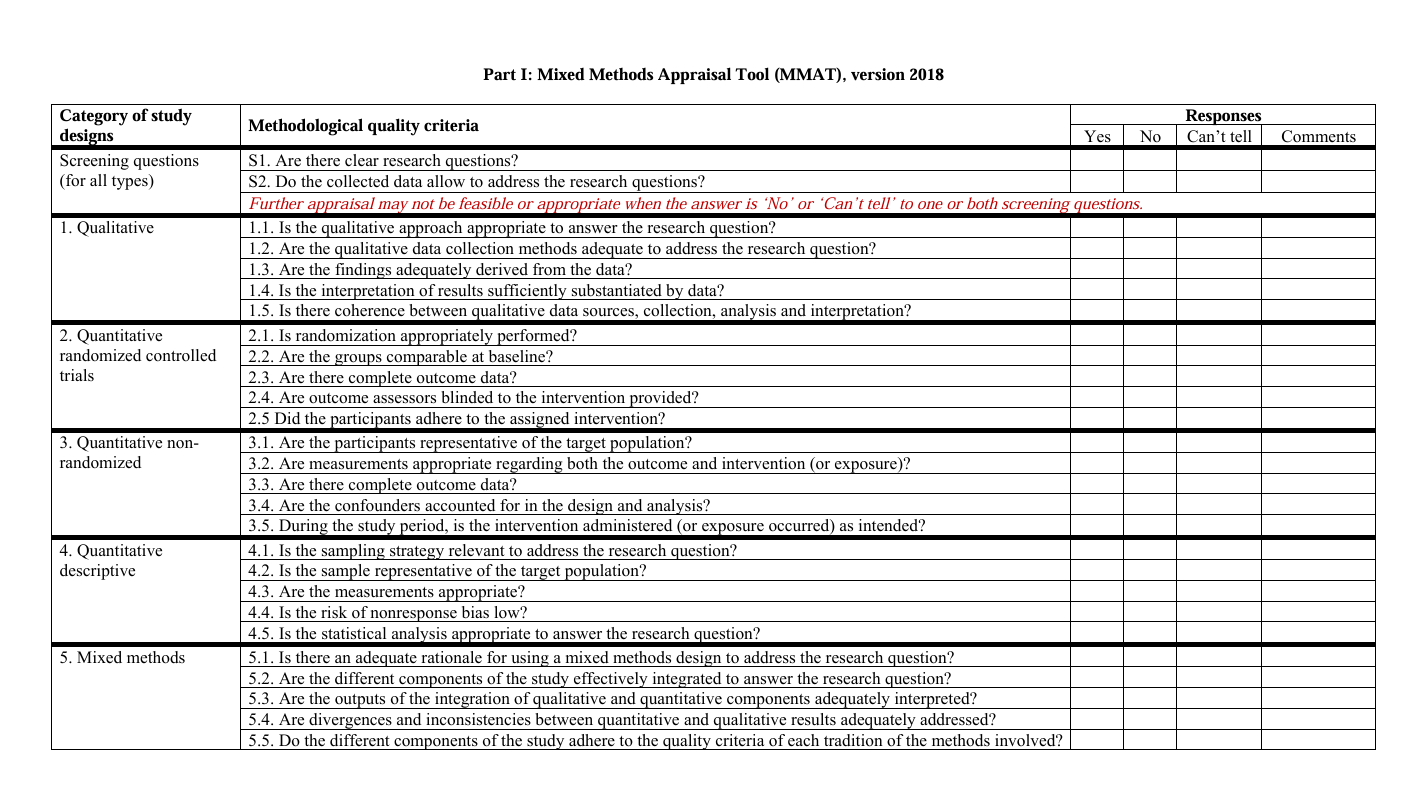


Supplementary material 3: Copy of Mixed Methods Appraisal Tool

Supplement: Copy of MMAT questions [file mmc3.docx]

Supplementary material 6:

**Funnel plot**


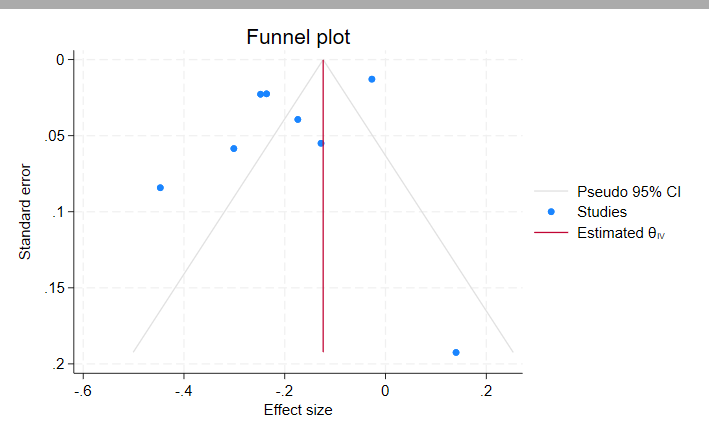

Supplement: Funnel plot [file mmc6.docx]
