# Supplementary material for: The inverse palliative care law in advanced lung disease: a mixed-methods systematic review and meta-analysis
Source: eClinicalMedicine. 2025 Dec 17;91:103697. doi: 10.1016/j.eclinm.2025.103697 (PMC12770954; doi:10.1016/j.eclinm.2025.103697)
Supplement: Details of all included studies [file mmc4.docx]

**Supplementary material 4**.

**Summary table of all included studies**

(Abbreviations: SPC= Specialist palliative care, EOL= End-of-life, SEP= Socio-economic position, OR= odds ratio, adj= adjusted for covariables, 95%CI= Confidence Interval). Green boxes highlighting the studies where SEP was the primary focus of the study.

| Study details  (Author, year, country) | Method | Population (Type of lung disease & number) | Measure of exposure (Socio-economic position) | Comparator (which measure of access or receipt or experience of palliative care) | Was variation in palliative care due to **SEP** the primary focus of the study? | MMAT  (% of quality criteria met) | Summary of Results socio-economic position (SEP) |
| --- | --- | --- | --- | --- | --- | --- | --- |
| 1. Vranas^40^   (2020, USA) | Quant (Retrospective cohort) | 23,142 with lung cancer | Individual income | Receipt of SPC | No | 100% | In the lowest income group, 55% received SPC, compared to 58% of the highest income group. No further statistical testing as SEP was not the focus of the study |
| 1. Hui^41^   (2005, Australia) | Quant  (Retrospective cohort) | 526 with lung cancer | Area level deprivation | Receipt of SPC | Yes | 60% | Reports no difference in receipt of palliative care due to SEP. No multivariable analysis. The comparison was residents of one half of Sydney (more affluent) compared to the other half (less affluent), rather than deprivation quintiles which is used in most other studies. |
| 1. Davidoff^42^   (2021, USA) | Quant  (Retrospective cohort) | 26,712 with lung cancer | Area level poverty rate | Receipt of symptom management or hospice | No | 100% | Reports that Medicare beneficiaries who died of lung cancer were more likely to receive symptom control and/or hospice rather than cancer-directed therapies, the closer they were to death. No further statistical testing related to SEP, as not the focus of the study. |
| 1. Sullivan^43^   (2017, USA) | Quant  (retrospective cohort) | 21,860 with lung cancer | Individual  income | Receipt of Hospice | No | 100% | With the lowest income group as the reference (1.00), those of moderate income had an increase in odds of hospice enrolment (adj OR 1.12; 95% CI 1.04-1.20). |
| 1. McCarthy^44^   (2003, USA) | Quant  (retrospective cohort) | 62,117 with lung cancer | Area-level income | Receipt of Hospice | No | 100% | In the lowest income quartile only 23 % accessed hospice, adj hazard ratio 0.95 (95%CI 0.90-1.00), compared to the highest income quartile, where 29% accessed hospice, adj hazard ratio 1.05 (95%CI 0.99-1.01) |
| 1. Simone^45^   (2012, USA) | Quant  (Prospective questionnaire) | 82 with lung cancer, 8 with pleural mesothelioma | Individual education level | Self-reported pain control | No | 60% | Those with a higher education level (66%) used analgesia for pain control more than those of a lower educational level (57%), however, this was not statistically significant (p=0.37). Although small sample size. 56% of patients said they did not take analgesia because they could not afford to pay for pain medication. |
| 1. Yan^46^   (2023, Canada) | Quant (Prospective cohort) | 13,159 with lung cancer | Area-level income | Receipt of palliative care | No | 100% | Those in the lowest income quintile were less likely to receive palliative care (adj OR 0.973; 95% CI 0.949-0.998) than those of higher income (Q5, reference= 1.00) |
| 1. Hardy^47^   (2011, USA) | Quant (Retrospective cohort) | 117,894 with lung cancer | Area-level poverty | Receipt of hospice services | Yes | 100% | Those in the lowest SEP quartile were 7% less likely to receive hospice care (adj OR 0.93; 95%CI 0.86-1.00) compared to the highest SEP quartile. |
| 1. John^48^   (2014, USA) | Quant (Prospective patient survey) | 4,334 with lung cancer | Individual level education & income | Perceived unmet needs including pain control | No | 100% | This study states that those in the lowest income quartile 11.4% had unmet care needs (including poor pain control and need for psychological support), compared to 7.2% in the highest income quartile (P<0.001). No further data related to SEP is reported. |
| 1. Bylicki^49^   (2021, France) | Quant (Retrospective cohort) | 79,746 with lung cancer | Area-level deprivation | Access to palliative care | No | 80% | Data on socio-economic position and receipt of palliative care were gathered, however, this was not published in the study. |
| 1. Mack^50^   (2013, USA) | Quant (retrospective cohort) | 52,710 with lung cancer | Area-level income | Receipt of hospice | Yes | 100% | Medicare patients aged over 65, using the highest income quartile as a reference (1.00), those in the lowest income quartile had a reduced odds of receiving hospice care (adj OR 0.83; 95% CI 0.77-0.90 in California and adj OR 0.82; 95% CI 0.75-0.90 in New York). |
| 1. Huskamp^51^   (2009, USA) | Quant (Prospective cohort) | 1,572 with lung cancer | Individual-level income and education | Discussing hospice & receipt of hospice | No | 100% | Medicaid patients (low income) were less likely to have had hospice discussed (50%) than those with Medicare (52%) or private health insurance (69%), p=0.03. Those who had discussed hospice were more likely to receive it. However, once adjusted for covariates, this was no longer statistically significant. |
| 1. Keating^52^   (2006, USA) | Quant (Retrospective cohort) | 2,646 with lung cancer | Area-level income  Area-level education | Receipt of hospice | No | 80% | Those in the lowest income quartile (area level) were less likely to use hospice than the higher income groups (p<0.001), no odds ratios reported and no adjustment for other variables. |
| 1. Huo^53^   (2019, USA) | Quant (Retrospective cohort) | 69,414 with lung cancer | Area-level poverty | Receipt of palliative care | No | 100% | Those living in the most affluent area had an increased odds (Reference= 1.00) of receiving palliative care compared to those in the most deprived adj OR 0.84; 95% CI 0.78-0.91, p<0.0001. |
| 1. Ding^54^   (2021, Australia) | Quant (Retrospective cohort) | 17,816 with lung cancer | Area-level deprivation | Setting of palliative care received | No | 100 | Those in the most disadvantaged groups were less likely to receive inpatient specialist palliative care (adj OR 0.66; 95%CI 0.55-0.80) than those in the more affluent groups. |
| 1. Shugarman^55^   (2008, USA) | Quant (Retrospective cohort) | 13,120 with lung cancer | Area-level income | Receipt of hospice | No | 100% | The focus of this study was to examine variation due to age and gender. Area-level income was gathered. However, as this was not the outcome of interest, statistical testing was not conducted for this. |
| 1. Chang^56^   (2022, USA) | Quant (Retrospective cohort) | 25,121 with lung cancer | Area-level income | Receipt of palliative care | Yes | 100% | Using the most affluent income group as a reference (1), the lowest income group had reduced odds of accessing SPC 0.639 (95% CI 0.542-0.754). |
| 1. Khullar^57^   (2022, USA) | Quant (Retrospective cohort) | 83,175 with lung cancer | Area-level income  Area-level education | Receipt of palliative care | Yes | 100% | In contrast to other studies, this study reports that patients in the high-income group had a higher risk of not receiving palliative care, adj HR 1.03; 95% CI 1.01-1.04 (low-income group as reference). However, the area with the highest educational level (a different marker of higher SEP) had the lowest risk of not receiving palliative care (adj HR 0.96; 95%CI 0.94-0.97). Therefore, the two measures of high SEP show opposite results when considering risk of not receiving palliative care. |
| 1. Nayar^58^   (2014, USA) | Quant (Retrospective cohort) | 91,039 with lung cancer | Area-level socio-economic status | Hospice enrolment | No | 100% | Receipt of hospice varied across the social gradient.  Those in the lowest SES quintile were less likely to receive hospice (adj OR 0.93) compared to the high/very high-income group (OR 1), P<0.05 (no confidence intervals provided). |
| 1. McLouth^59^   (2023, USA) | Quant (prospective survey) | 77 with lung cancer | Individual education level | Receipt of palliative care | No | 60% | Although educational level was recorded, this was not included in the full statistical analysis The most common major barrier to patients being willing to accept palliative care was concern that their health insurance would not cover it (28.6% of respondents) |
| 1. Goldie^60^   (2021, Canada) | Quant (Retrospective cohort) | 37,203 with lung cancer | Area-level income | Receipt of early palliative care | No | 100% | All patients had advanced non-small cell lung cancer, 56% did not receive palliative care. SES was gathered, but variation in receipt of SES based on this was not calculated, as this was not the aim of the study. |
| 1. Schweiger^61^   (2023, USA) | Quant (Retrospective cohort) | 23142 with lung cancer | Individual income | Receipt of palliative care | No | 100% | Of those who did **not** receive palliative care, 35% were in the lowest income group and 33% in the highest income group.  However, this was not the outcome of interest, so no further testing was conducted. |
| 1. Huo^62^   (2021, USA) | Quant (Retrospective cohort) | 78473 with lung cancer | Area-level poverty | Receipt of palliative care | No | 100% | The focus of the study was variation in access to palliative care between US states. Raw data given for receipt of SPC, but no further analysis related to SEP, as not the primary focus of the study. |
| 1. Saphire^63^   (2020, USA) | Quant (Retrospective cohort) | 16246 with lung cancer | Area-level poverty | Receipt of medications for symptom control | No | 100% | People living in the lowest poverty area (i.e. the most affluent), experienced a statistically significant increase in medications for pain control. |
| 1. Burt^64^   (2010, UK) | Quant (Cross-sectional survey) | 252 with lung cancer | Area-level deprivation | Receipt of palliative care | No | 80% | Overall, 39.3% of patients accessed palliative care. Small sample and disproportionate amount from the lowest SEP group, so no statistically significant difference between groups. No multivariable analysis related to SEP as not the focus of the study. |
| 1. Kendzerska^65^   (2019, Canada) | Quant (Retrospective cohort) | 15638 with lung cancer (+24082 with lung cancer and COPD) | Area-level income | Receipt of palliative care within the final 90 days of life and place of death | No | 100% | Those with lung cancer and/or COPD (mixed group), with lower SEP were less likely to receive palliative care compared to those of higher SEP. With the lowest income quintile as a reference (1.00), the highest income quintile had an adj OR 1.64 (95%CI 1.60-1.68) of receiving a palliative medicine physician home visit and adj OR 1.56 (95%CI 1.52-1.60) of palliative home care.  Those with lung cancer, with or without COPD were significantly more likely to receive home SPC compared to those with COPD only. Adjusted OR 4.22 (95%CI 4.08-4.37) to receive palliative care, compared to adj OR 0.82 (0.81-0.84) If COPD alone. Those with COPD had lower income status and were more likely to die in hospital. |
| 1. Strang^66^   (2021, Sweden) | Quant (Retrospective cohort) | 3562 with lung cancer +2917 with COPD | Area level deprivation | Receipt of SPC | No | 100% | The likelihood of receiving specialist palliative care during the final 3 months of life for people with lung cancer or COPD was significantly higher for those of higher SEP.  With the lowest SEP group as a reference (1.00),  the highest SEP group adj OR 1.33 (95% CI 1.14-1.56) of receiving SPC.  However, when the analysis was done separately for people with COPD- there was no statistically significant difference between SEP groups (for those with lung cancer or lung cancer with COPD the higher SEP group had increased odd of receiving SPC compared to the lowest SEP groups). |
| 1. Fairlamb^67^   (2021, UK) | Qual | 16 healthcare professionals interviewed | No direct measures, includes discussion of “socio-economic” factors | Barriers to receiving SPC | No | 100% | Participants reported on the impact of socioeconomic position on public perception of the disease. COPD being more prevalent in lower socioeconomic classes was thought to lead to people not appreciating the extent of the disease burden. It also reports that there is stigma associated with COPD and blame due to smoking. |
| 1. Rush^68^   (2017, USA) | Quant (Retrospective cohort) | 181,689 with COPD | Area-level income | Receipt of SPC | No | 100% | Those of higher SEP, had increased odds of receiving palliative care than those of lower SEP.  1^st^ quartile (lowest income)- Reference (1.00)  2nd quartile adjusted OR 1.19 (95%CI 1.07-1.32)  3rd quartile adjusted OR 1.20 (95%CI 1.08-1.34)  4th quartile (highest income) adjusted OR 1.41 (95%CI 1.26-1.58), P<0.01 |
| 1. Carlucci^69^   (2016, Italy) | Mixed methods | 72 with COPD | Individual education level | Patient preference for palliative and end-of-life care | No | 100% | Multivariate logistic regression analysis compared patients' EOL choices and demographics. Patients who chose Intubation over symptom control (despite very severe COPD) were more likely to have a lower educational level, adj OR 15.71 (95%CI 1.65-402.41). The strongest predictor of choosing palliative care was higher educational level adj OR 12.94 (95%CI 1.98-259.38). |
| 1. Knauft^70^   (2005, USA) | Mixed methods | 115 patients with COPD  + 56 healthcare professionals | Individual income  Individual educational level | Patient preference and barriers to discussing end-of-life care | No | 60% | This study reported that only 1/3rd of patients with oxygen-dependent COPD had discussions about the future and wishes for EOL care.  Patient demographics, including SEP measured, but comparisons between SEP and preferences for palliative and end-of-life care were not made. |
| 1. Fu^71^   (2021, UK) | Qual | 20 patients with COPD,  6 carers,  25 healthcare professionals | Individual educational level | Views on barriers to accessing palliative care | No | 100% | 80% of patients and carers interviewed only had education up to GCSE level. The term “palliative care” was felt to be a barrier to access, as participants has a misperception that palliative care was only for patients with a cancer diagnosis (and not COPD). Some patients/carers did not access hospice services due to distance to travel. |
| 1. Chou^72^   (2017, Taiwan) | Quant (Prospective cross-sectional) | 101 patients with COPD | Individual educational level | Patient preference/willingness to accept palliative care | No | 80% | This study explains that multiple linear regression was used to test demographic factors affecting patients’ willingness to accept palliative care and that higher educational level significantly predicted willingness to accept palliative care. However, full data is not shown. |
| 1. White^73^   (2011, UK) | Qual | 163 patients with COPD | Area-level deprivation and individual level education | Palliative care needs and preferences for place of care. | No | 100% | Most patients were in the lower SEP groups and had low educational attainment. It was identified that patients wanted to improve their symptoms, especially breathlessness, with 40% describing themselves as profoundly incapacitated. Patients often had multiple hospital admissions for exacerbation of COPD; however, they said they would still want to be readmitted again if they had a further exacerbation (Avoidance of hospital admission was not a priority). |
| 1. Scheerens^74^   (2020, Belgium) | Quant | 58527 with COPD | Individual level income and educational level | Receipt of palliative care at home | No | 100% | Although details on SES were gathered, this was used in analysis as a potential confounding factor. The analysis reports the association between receiving home palliative care and reduced hospital admissions and the impact on cost savings.  Only 3% of those who died of COPD received SPC homecare. Those who accessed SPC at home were hospitalised significantly few times (24.7% vs 47.9%) and were more likely to also be inpatient SPC. |
| 1. Gershon^75^   (2018, Canada) | Quant | 151912 patients with COPD | Area-level income | Receipt of specialist palliative care | No | 60% | This study reports that 2/3rds of people living in Ontario between 2004-2014 were of lower to mid socio-economic status. In 2004, 5.3% accessed SPC per year, which increased to 14.3% by 2014. Showing some improvement in people with COPD accessing palliative care. However, the relationship between access to palliative care and SEP is not reported. |
| 1. Sono^76^   (2023, Thailand) | Quant | 280 patients with COPD | Individual level education & income | Awareness of what palliative care is | No | 40% | In this study, 73.2% of participants were in the lowest income group and 60.4% were of low education (primary school level or less).  Overall, only 22.6% of patients were aware of palliative care. There was no analysis of whether there was a relationship between SES and palliative care awareness. |
| 1. Rush^77^   (2018. USA) | Quant | 3166 with ILD | Area-level income | Receipt of SPC | No | 100% | 3166 patients with pulmonary fibrosis admitted for mechanical ventilation, 408 (12.9%) received SPC. Those of higher SEP were more likely to receive specialist palliative care.  Lowest income quartile -Reference (1.00)  Highest income quartile adj OR 1.33 (95%CI 0.94-1.87) |
| 1. Cross^78^   (2020, USA) | Quant (Retrospective cohort) | 2026758 with COPD + 246210 with ILD | Area-level educational level | Place of death, including hospice | No | 100% | From 2003-2017 there was an increase in patients with COPD dying at home. There was also an increase in patients with COPD dying in hospice. Patients with ILD continued to die in hospital.  Those in the higher educational group has a reduced odds of death in hospital (adjusted OR 0.97; 95%CI 0.97-0.98) compared to the lower educated who were more likely to die in hospital. The highest educated had an increased odds of death at home (adjusted OR 1.03; 95%CI 1.01-1.04) or hospice (adjusted OR 1.19; 95%CI 1.16-1.22). These differences were statistically significant (<0.001). |
| 1. Higginson^79^   (2017, UK) | Quant (Retrospective cohort) | 334520 with COPD + 45712 with ILD | Area-level deprivation | Place of death | No | 100% | Having co-morbidities and living in deprived areas independently increased the chance of dying in hospital, with larger effects for ILD (Proportion Ratios 1.01–1.55) than COPD (PRs 1.01–1.39) and a “dose–response” relationship. (PR= Proportion Ratio)  Hospital was the most common place of death (67.3% for COPD, 70.1% IPD), followed by home (19.9% COPD, 19.1% IPD). Deaths within hospices accounted for just 0.9% of COPD and 2.9% of IPD cases. |
| 1. McVeigh^80^   (2019, USA) | Qual | 16 healthcare professionals | No direct measures, includes discussion of “socio-economic” factors | Barriers to accessing SPC | No | 100% | Healthcare professionals reported that patients (particularly veterans) with COPD/ILD may not be able to fully access palliative care services due to financial reasons: “*I don't think it's quite as easy to provide everything that they [veteran population with lung disease] need to maintain their quality of life, because we do whatever they need for oxygen, we're kind of in charge of that but out in the real world [outside the veteran hospital] it's run by what insurance wants to pay and what the patient ultimately can pay too*.” |
| 1. Penn^81^   (2014, USA) | Quant (Retrospective cohort) | 76074 with lung cancer | Area-level education | Intervention aiming to improve access to SPC | No | 100% | The lower the SEP (based on education level), the less likely the patient was to access hospice.  With highest educational quartile as the reference (1.00),  the lowest educational quartile adj OR 0.78; 95%CI 0.75-0.82 of accessing hospice. |
| 1. Scheerens^82^   (2020, Belgium) | Quant (non-blinded RCT) | 70 with COPD | Individual level education | Intervention aiming to improve access to SPC | No | 40% | SEP gathered at baseline. No analysis related to SEP. |
| 1. Horton^83^   (2013, Canada) | Quant (Cohort observational) | 30 patients with COPD + 18 carers | Individual income | Intervention aiming to improve access to SPC | No | 60% | SEP gathered at baseline. No analysis related to SEP. |
| 1. Nguyen^84^   (2018, USA) | Quant (prospective quasi-experimental design) | 202 with lung cancer + 122 carers | Individual income and education | Intervention aiming to improve access to SPC | No | 60% | SEP gathered at baseline. No analysis related to SEP. |
| 1. Ferrell^85^   (2015, USA) | Quant (prospective quasi-experimental design) | 491 with lung cancer | Individual income and education | Intervention aiming to improve access to SPC | No | 80% | SEP gathered at baseline. No analysis related to SEP. |
| 1. Iqbal^86^   (2020, Canada) | Quant (prospective observational cohort) | 38851 with lung cancer | Area-level income | Delphi criteria aiming to improve access | No | 100% | Those in the lowest income quintile had the highest percentage of patients found to be *eligible* for SPC (24.5% in quintile 1 Vs 16.1% in quintile 5), i.e. the lower the income the higher the need for palliative care.  Amongst those patients who were *eligible* for SPC,  with those in the highest SEP group as the reference (1.00),  those in the lowest SEP group has adj OR 1.13 (95%CI 1.08-1.18) of receiving SPC. |
| 1. Reilly^87^   (2023, UK) | Mixed methods (randomised feasibility trial) | 31 COPD  + 5 ILD  + 2 lung cancer | Area-level deprivation + individual education level | Intervention to improve palliative care | No | 80% | SEP gathered at baseline. No analysis related to SEP. |
| 1. Armstrong^88^   (2025, USA) | Quant  (retrospective cohort study) | 63 lung cancer | Area-level income | Receipt of SPC | No | 80% | Data gathered but not reported, as sample too small with too little variation between categories |
| 1. Madiraca^89^   (2024, USA) | Mixed methods | 30 COPD | Individual level education | Barriers to SPC/symptom control | No | 40% | A major theme from qualitative interviews was symptom control affected by being unable to access medicine/medical equipment due to cost. Some (40%) of women reported lack of medication for their symptoms due to insurance barriers and 53% reported issues accessing oxygen.  None of the patients had received palliative care and 80% did not know what palliative care means. |
| 1. Temel^90^   (2024, USA) | Quant (Randomise controlled trial) | 497 with lung cancer & 10 with pleural mesothelioma | Individual income and education level | Intervention- comparing stepped vs early SPC | No | 80% | SEP at baseline to show groups are similar and included those of lower SEP in both. One group early SPC, the other stepped SPC. QoL scores similar but stepped palliative care had significantly less visits per patients (p<0.01) and so would be a more sustainable use of resource.  No comparisons made by SEP or specific aims to increase access for low SEP patients. |
| 1. Edmonds^91^   (2025, USA) | Quant (prospective cohort study) | 99 with lung cancer | Individual education and individual income | Receipt and preferences for SPC | No | 100% | Data focuses on whether those from ethnic minority groups accessed palliative care compared to non-minority ethnic groups. SEP was primarily gathered as a co-variable to adjust for. |
| 1. Landers^92^ (2024, New Zealand) | Qual | 24 with COPD  +14 carers  + 30 healthcare professionals | No direct measures, includes discussion of “socio-economic” factors | Barriers to palliative care | No | 80% | The study reports inequity within the healthcare system, with services designed for people with financial resources to access care. Participants with end-stage COPD reported that they needed to use public transport to attend appointments and that the cost (and lack of available) public transport was a barrier. Patients could not afford the $47 (NZD) charge to attend General Practice appointments, leading to attendance at emergency departments for end-of-life care. Funding was not available for equipment such as wheelchairs which had a negative impact on quality of life for those without financial resources to buy them themselves. |
| 1. Wakefield^93^   (2025, UK) | Quant (Retrospective cohort) | 151 with mesothelioma | Area-level deprivation | Receipt of SPC | No | 80% | This study reports 69% of patients with mesothelioma had at least 1 unplanned hospital admission in the final 90 days of life, with 20% having 3+ admissions. Those of higher socio-economic position had less admissions on average, but this was not statistically significant (small sample size). Specialist palliative care was received by patients, at home 34%, in hospital 26% and hospice 11%. |
