# Supplementary material for: The inverse palliative care law in advanced lung disease: a mixed-methods systematic review and meta-analysis
Source: eClinicalMedicine. 2025 Dec 17;91:103697. doi: 10.1016/j.eclinm.2025.103697 (PMC12770954; doi:10.1016/j.eclinm.2025.103697)
Supplement: Sensitivity analysis and GRADE [file mmc5.docx]

**Supplementary material 5:**

**Leave-one-out-sensitivity analysis**

**Leave-one-out sensitivity analysis was conducted to quantify the impact of each study on the estimation of effect size. The table below shows that leaving out any one study, did not lead to a large change in the estimation of effect size, which suggests that our findings are robust.**

| Omitted study | E.S (95% CI) |
| --- | --- |
| Chang2022 | 0.84 (0.78-0.92) |
| Hardy2011_urban | 0.83 (0.74-0.93) |
| Hardy2011_rural | 0.81 (0.74-0.89) |
| Huo2019 | 0.82 (0.73-0.92) |
| Mack2013_CA | 0.81 (0.73-0.91) |
| Mack2013 NY | 0.83 (0.75-0.92) |
| Penn 2014 | 0.83 (0.74-0.93) |
| Yan 2023 | 0.79 (0.74-0.85) |
| All studies included | 0.82 (0.75-0.90) |

**GRADE of evidence^39^**

The GRADE (Grading of Recommendations, Assessment, Development and Evaluation) approach was used to assess the certainty of evidence.

| A | B | C | D | E |
| --- | --- | --- | --- | --- |
| Type of study | Initial certainty of evidence (CofE) | Five domains which can downgrade CofE | Three domains which can upgrade CofE | Resulting GRADE of certainty of evidence |
| Non randomised control trials | HIGH (as study type appropriate)    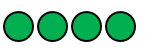 | 1. **Study limitations**: No serious concerns (0) 2. **Inconsistency:** No serious concerns (high heterogeneity, but leave-one-out sensitivity analysis shows robustness of E.S) (0) 3. **Indirectness**: No concerns about applicability, external validity, or translatability (0) 4. **Imprecision:** No serious concerns (0) 5. **Dissemination bias**: Possible concerns based on funnel plot (although number of studies is low)   = **DOWNGRADE (-1)**    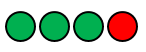 | 1. Large effect size (0) 2. Dose response gradient (0) 3. Opposite residual confounding (0) | HIGH 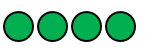  MODERATE 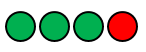 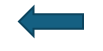  LOW 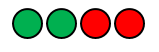  VERY LOW 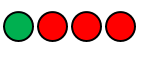  Final rating= **MODERATE** |
